# Note from the Editor: *EHP* International Program

**Published:** 2008-07

**Authors:** Hugh A. Tilson

**Affiliations:** *EHP*, E-mail: EHPEditor@niehs.nih.gov

We at *EHP* are proud of our work to bring environmental health science to a global audience. This month we are pleased to introduce a new, expanded website that provides information on our International Program, outlines our international outreach activities, and allows readers to easily access all the *EHP* content that has been translated into other languages. Come visit our International Program website at www.ehponline.org/international and let us know what you think.

